# Real-world 12-month outcomes of Risdiplam in spinal muscular atrophy types 2 and 3: A Brazilian cohort

**DOI:** 10.1016/j.clinsp.2026.101027

**Published:** 2026-07-18

**Authors:** Clara Gontijo Camelo, Rodrigo Holanda Mendonça, Cristiane Araujo Martins Moreno, Graziela Jorge Polido, Eduardo Vital de Carvalho, Weverton Carlos da Silva Teixeira, Daniel Shoji Hayashi, Joemir Jabson da Conceição Brito, Ciro Matsui Junior, Edmar Zanoteli

**Affiliations:** Department of Neurology, Faculdade de Medicina, Universidade de São Paulo (FMUSP), São Paulo, SP, Brazil

**Keywords:** SMA, Spinal muscular atrophy, Risdiplam, SMA type 2, SMA type 3, Real-world

## Abstract

**Introduction:**

Spinal Muscular Atrophy (SMA) is an autosomal recessive disorder characterized by progressive axial and proximal limb weakness, often leading to ventilatory insufficiency. Risdiplam, an oral SMN2 splicing modifier, has demonstrated efficacy across clinical trials involving both infantile and late-onset SMA. However, real-world data on its long-term safety and effectiveness remain limited.

**Objective:**

To evaluate the safety and preliminary efficacy of Risdiplam after one year of treatment in patients with SMA types 2 and 3.

**Methods:**

This retrospective, single-center study included patients with genetically confirmed SMA treated with Risdiplam at the Neuromuscular Clinic of the Hospital das Clínicas, University of São Paulo, Brazil. All participants were treatment-naïve before initiating Risdiplam and were followed for 12-months. Motor function (CHOP-INTEND, HFMS), pulmonary function (FVC, FEV₁), and necessity of G-tube were assessed at baseline, 6 m, and after one year of therapy.

**Results:**

Sixteen patients were included (mean age 20-years, range 6–56). Eleven patients (69%) had SMA type 2, while 5 (31%) had SMA type 3. The mean untreated disease duration was 18-years (range 5–44). After 12-months, 13-patients (81%) showed apparent stabilization or improvement in motor function, while 3 (19%) exhibited motor decline. Pulmonary function improved or stabilized in 13 patients (81%) and declined in 3 (19%). Clinical decline was observed in patients with progressive scoliosis or overweight. All patients maintained exclusive oral feeding.

**Conclusion:**

Preliminary data from the one-year follow-up suggest that Risdiplam is safe and appears generally effective in preserving or enhancing motor, respiratory, and swallowing functions. Patients with severe skeletal deformities exhibited less favorable outcomes

## Highlights

• Risdiplam stabilized or improved motor and respiratory function in patients with SMA types 2/3.
• Severe scoliosis or obesity negatively impacted functional outcomes despite treatment.
• Risdiplam was well tolerated; diarrhea was the most common adverse event.
• This real-world evidence supports the effectiveness of oral risdiplam in late-onset SMA.

## Introduction

Spinal Muscular Atrophy (SMA) is an autosomal recessive genetic disease of lower motor neurons from the spinal cord and motor nuclei of the brainstem, leading to a progressive axial and proximal limb weakness associated with ventilatory insufficiency.^1^ The most common form of SMA is caused by deletions or disease-causing variants in the Survival Motor Neuron 1 (SMN1) gene located on chromosome-5 (SMA-5q) (1). Around 1 in 11,000 people are affected by the disorder.^2^

SMA-5q patients are typically classified into at least three clinical forms based on their maximum motor ability and age of onset. Type 1 SMA-5q is the most common form and is characterized by an early onset (0‒6 months of age), and the children do not acquire the ability to sit unaided. Type 2 SMA-5q manifests between 6- and 18-months of age, and affected children are unable to walk unaided. Type 3 SMA-5q typically starts after the second year of life, and affected individuals can walk unaided.^2^^,^^3^

The disease is caused by a deficiency of the Survival Motor Neuron (SMN) protein, which is encoded by two nearly identical genes: SMN1 and SMN2, both located on chromosome 5.^1^^,^^3^, ^4^, ^5^ A critical single-nucleotide substitution in exon 7 of SMN2 results in aberrant splicing, leading to predominant exclusion of exon 7 during mRNA processing.^1^^,^^3^ Consequently, *SMN2* primarily produces a truncated, unstable SMN protein, with only ∼10% of transcripts yielding a functional protein.^3^^,^^4^ The copy number of SMN2 serves as a key phenotypic modifier, with higher copy numbers generally associated with milder clinical presentations.^2^

In recent years, transformative therapeutic strategies have become available for SMA. These include gene replacement therapy with SMN1 (onasemnogene abeparvovec, AVXS-101) and SMN2 splicing modifiers (nusinersen, risdiplam).^6^, ^7^, ^8^, ^9^, ^10^, ^11^ Risdiplam is an orally administered small molecule that functions as an SMN2 splicing modifier that promotes inclusion of exon 7 in SMN2 transcripts, correcting the splicing of SMN2 pre-mRNA and thereby increasing the production of functional Survival Motor Neuron (SMN) protein in patients with SMA.^7^ The medication is approved in many regulatory agencies in the world, including in Brazil.

Risdiplam has demonstrated efficacy across pivotal clinical trials, including infantile and late-onset SMA patients,^7^^,^^9^^,^^12^, ^13^, ^14^, ^15^ but real-world, post-marketing evidence regarding its safety and effectiveness remains limited.^16^^,^^17^

This study aims to present data on the safety and efficacy of Risdiplam in patients who have undergone treatment for a duration of one year.

## Methods

### Study design and participants

This was a retrospective, single-center study of patients with genetically confirmed Spinal Muscular Atrophy (SMA) treated with Risdiplam with clinical diagnosis of SMA types 2 or 3. All patients were treatment-naïve before initiating Risdiplam and were followed for at least 12-months at the Neuromuscular Clinic of the Hospital das Clínicas, University of São Paulo School of Medicine, Brazil. Access to Risdiplam occurred either through the public health system, health insurance, or through legal action. Patients underwent evaluations at baseline (before Risdiplam initiation) and subsequently at 6- and 12-months of treatment. Written informed consent was obtained from all patients and/or legal guardians. The study protocol was approved by the local ethics committee (87773118.7.0000.0068)

### Motor function assessment

Motor outcomes were evaluated using the Children’s Hospital of Philadelphia Infant Test of Neuromuscular Disorders (CHOP-INTEND; range 0–64) in younger or non-sitting patients, and the Hammersmith Functional Motor Scale (HFMS; range 0–66) in older or sitting patients.^18^^,^^19^ Motor assessments were performed by one unblinded exclusive evaluator.

### Pulmonary function assessment

Pulmonary function was assessed by percent-predicted Forced Vital Capacity (FVC) and absolute Forced Expiratory Volume in one second (FEV₁) in patients capable of completing the respiratory tests. In addition, the requirement and pattern of non-invasive ventilation and the frequency of hospitalizations due to respiratory infections were recorded. Respiratory assessments were performed by one unblinded exclusive evaluator.

### Bulbar function assessment

Bulbar function was evaluated based on the indication for gastrostomy and the presence of aspiration or choking episodes.

### Statistics

Descriptive statistics were reported as mean ± SD, median (interquartile range), or frequency (%).

## Results

**Fig. 1:**
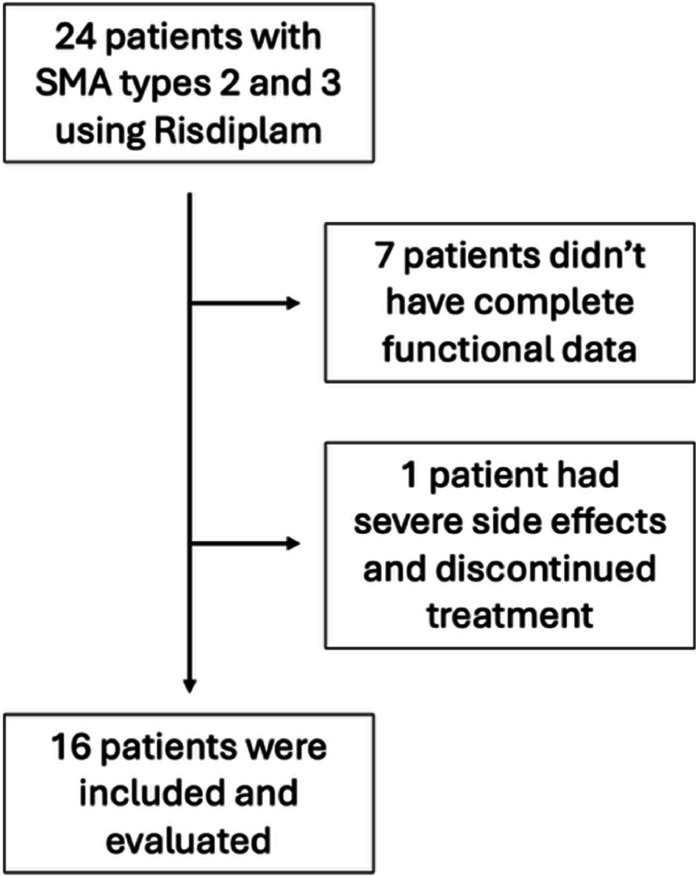
Patient selection flowchart.

A total of 24 patients with SMA-5q types 2 and 3 who were receiving Risdiplam and had not previously been treated with any disease-modifying therapy were initially enrolled. Seven patients were excluded because they presented inconsistent assessments (didn’t present both motor and respiratory data, or lack of assessments at 6- and/or 12-months)**.** One additional patient discontinued treatment after three months owing to severe gastrointestinal adverse events, including nausea, vomiting, diarrhea, and abdominal distension. A total of 16 patients were included in this analysis (Fig. 1).

Fourteen patients (87.5%) carried three SMN2 copies and 2 (12.5%) carried four SMN2 copies. Five patients (31%) presented with SMA type 3 phenotype and had achieved independent ambulation, while 11 (69%) exhibited an SMA type 2 phenotype and had acquired the ability to sit without support. Only one patient carried a point mutation in a single SMN1 allele; all others had exon 7 homozygous deletions in SMN1*.*

The mean age at treatment initiation was 20-years (range: 6–56), and the mean age of disease duration without treatment was 18-years (range 5‒44) (Table 1).

**Table 1:** Clinical, genetic, and functional characteristics of the patients.

Patient	Type of SMA	SMN2 copies	Homozygous	Symptoms (rge of treatmessease duratiolatory funct				Bulbar	HMSE basal	HMSE 6m	HMSE 12m	CHOP basal	CHOP 6m	CHOP 12m	Summary
P1	2	3	Yes	6	12	12	Bipap Not	Oral	NA	NA	NA	45	39	35	Declined
P2	3	3	Yes	14	6	5	No	Oral	45	42	44	NA	NA	NA	Stable
P3	2	3	Yes	17	18	17	Bipap Not	Oral	NA	NA	NA	43	62	39	Declined
P4	2	3	Yes	6	11	11	Bipap Not	Oral	3	4	2	47	53	45	Stable
P5	2	3	Yes	8	18	18	Bipap Not	Oral	3	3	38	41	41	54	Improved
P6	3	4	Yes	84	17	13	No	Oral	43	48	47	NA	NA	NA	Improved
P7	2	3	Yes	12	7	6	No	Oral	29	25	26	NA	NA	NA	Declined
P8	2	3	Yes	7	10	10	Bipap Not	Oral	26	14	25	NA	NA	NA	Stable
P9	2	3	Yes	1	23	22	No	Oral	5	6	6	NA	NA	NA	Stable
P10	2	3	Yes	8	14	14	Bipap Not	Oral	NA	NA	NA	50	56	58	Improved
P11	2	3	Yes	1	33	33	Bipap Not	Oral	4	12	14	NA	NA	NA	Improved
P12	2	3	Yes	9	15	15	Bipap Not	Oral	4	4	3	43	44	44	Stable
P13	3	3	Yes	24	15	13	No	Oral	46	44	45	NA	NA	NA	Stable
P14	2	3	Yes	7	29	29	Bipap Not	Oral	NA	NA	NA	28	29	30	Stable
P15	3	3	Yes	36	39	36	No	Oral	26	27	28	NA	NA	NA	Stable
P16	3	4	Yes	144	56	44	No	Oral	41	43	46	NA	NA	NA	Improved

### Motor function

**Fig. 2:**
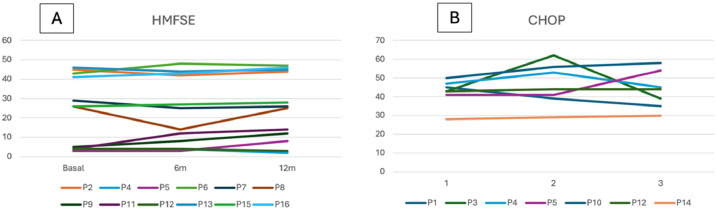
(A) HMFSE and (B) CHOP-INTEND in symptomatic patients at baseline, 6-months, and 12-months of Risdiplam treatment.

Twelve patients were evaluated only through the HMFSE scale, four patients were evaluated only through the CHOP-I scale, and three patients were evaluated using both scales (P4, P5, P12). Thirteen patients (81%) demonstrated improvement or stabilization of motor function (≤ 2-point variation on the motor function scale) (Fig. 2).^20^^,^^21^ Three patients (19%) experienced motor decline. On the HFMSE scale, the mean change from baseline was 2.1-points (range: −3 to 10), while on the CHOP-INTEND scale, the mean change from baseline was 1.1-points (range: −10 to 13). Five patients (P5, P6, P10, P11, and P16) demonstrated motor improvement of more than three points on the assessment scales (Table 1).

### Respiratory and bulbar function

**Fig. 3:**
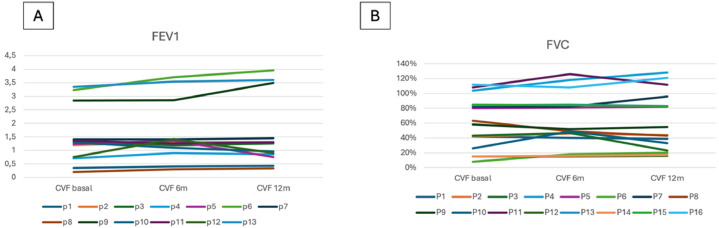
(A) FEV1 and (B) FVC in symptomatic SMA patients at baseline and after 6- and 12-months of risdiplam treatment.

Pulmonary function improved or stabilized in 13 patients (81%) and declined in 3 patients (19%) (Fig. 3).

Among the patients who experienced motor and respiratory decline, one individual (P1) presented with scoliosis with a Cobb angle of 50° at treatment initiation, which progressed to 70° after 12-months. Another patient (P3) initiated Risdiplam with a Cobb angle of 120° and was no longer a candidate for corrective surgery; although no further progression was observed after one year, the patient was already severely compromised at baseline. A third patient (P4) presented with obesity (BMI = 31.9) and scoliosis measuring 70° at baseline, which progressed to 107° after 12-months of treatment.

At baseline, 9-patients (64%) required nocturnal non-invasive ventilation, which remained necessary throughout the observation period. One patient (7%) had experienced eight pneumonia-related hospitalizations prior to treatment, but none thereafter. Two patients (14%) were hospitalized for pneumonia during the 12-month treatment period.

No patient required gastrostomy, and all maintained exclusive oral feeding throughout follow-up. One patient (7%) reported choking at baseline and was initially considered for gastrostomy tube placement; however, this intervention was no longer deemed necessary after initiation of treatment.

### Side effects

Adverse events were observed in three patients (17.7%). One patient developed severe diarrhea during the first month of risdiplam therapy, which necessitated discontinuation of the medication. Following drug withdrawal, the diarrhea resolved, and the patient elected not to resume treatment. Two additional patients also reported treatment-related diarrhea; however, in these cases, the symptoms were mild and did not require dose interruption or discontinuation.

## Discussion

This study represents the first Brazilian cohort to evaluate the efficacy and safety of Risdiplam in naive SMA patients.^10^^,^^22^

After one year of follow-up, treatment with Risdiplam was associated with apparent stabilization of motor and respiratory function in most patients, and functional gains in a subset of cases. Notably, this cohort was composed predominantly of symptomatic individuals with SMA types 2 and 3 who initiated therapy later in life, so most of these patients had a more chronic and stable disease.

Over the course of one year, patients with SMA types 2 and 3 typically experience a gradual annual decline in both motor and respiratory domains. Natural history studies have reported annual reductions in Forced Vital Capacity (FVC) ranging from 0.1% to 6.3% in untreated patients.^23^, ^24^, ^25^, ^26^, ^27^, ^28^ Thus, the observation of functional improvement in some individuals with longstanding disease is particularly noteworthy. In the pivotal FIREFISH study, even among patients who already exhibited clinical signs of neuronal degeneration, a subset demonstrated measurable motor improvements.^12^ Moreover, even in patients without measurable motor gains, improvements in quality of life were reported, including a reduced frequency of hospitalizations due to pulmonary infections.

In patients with long-standing disease, clinically meaningful improvement with treatment is not expected to occur in the short term. Moreover, this group of patients often presents with musculoskeletal abnormalities, such as scoliosis, which may limit potential functional gains. Therefore, maintaining long-term stability represents an important therapeutic objective. In our one-year cohort, the mean change from baseline on the HFMSE scale was 2.1-points, while the mean change on the CHOP-INTEND scale was 1.1-points. In the SUNFISH study, at month 24, the mean change from baseline in the HFMSE total score was 2.2-points in the group receiving Risdiplam, whereas in patients who initially received placebo, the mean change from baseline was 0.0 after 12-months of Risdiplam treatment.^29^ Comparison with this large study highlights the relevance of our cohort’s findings.

Despite receiving treatment, a subset of patients demonstrated deterioration in motor and respiratory function, which was accompanied by severe and progressive scoliosis. Although scoliosis and overweight may have contributed to the lack of treatment response in our study, these observations are subject to potential confounding by disease severity, age, and SMN2 copy number, given the observational nature of the data. This phenomenon has already been described in the literature, particularly among non-ambulatory patients with low HFMSE scores and only two or three SMN2 copies, who remain at higher risk for progressive scoliosis even under disease-modifying therapy.^30^, ^31^, ^32^, ^33^ Such progression complicates the interpretation of disease trajectory in certain cases, as scoliosis alone may account for functional decline. In these scenarios, the application of biomarkers becomes crucial for quantifying the degree of neuronal degeneration and for evaluating the expected benefit of transitioning to an alternative therapeutic strategy.^34^

Interestingly, the patient carrying four SMN2 copies still exhibited a substantial disease burden in this cohort and derived measurable benefit from Risdiplam therapy, underscoring the importance of including such patients in government-supported therapeutic programs.^35^

Risdiplam has the potential to induce off-target effects during splicing modulation or in attempts to replace the defective SMN1 gene.^36^ Such off-target activity is believed to contribute to Adverse Events (AEs),^36^, ^37^, ^38^ likely through misrecognition and non-specific interactions. However, in phase III clinical trials and post-marketing studies, the most frequently reported adverse events were diarrhea, urinary tract infections, and respiratory infections.^7^^,^^9^^,^^12^^,^^14^

In our study, one patient was excluded from the follow-up because of severe gastrointestinal side effects. Amongst those patients who were followed during one year, we observed diarrhea in two patients. This adverse effects was limited to the early stages of treatment. However, discontinuations due to adverse events may indicate tolerability issues and can lead to an underestimation of long-term safety risks if patients with unfavorable responses are no longer followed. Risdiplam-associated side effects appear to occur more frequently during the first month of therapy and, in some cases, after more than one year of continuous use.^39^ Therefore, long-term monitoring remains essential to ensure sustained safety.

This study has several limitations. The first is that it represents only a one-year evaluation of patients in a more chronic and stable phase of the disease, during which motor and respiratory functions tend to decline more slowly. The cohort was heterogeneous and sample size was small, as only patients, from a single-center who had received Risdiplam as their sole disease-modifying therapy were included, while those previously treated with other agents were excluded. This might have led to a potential selection bias. In Brazil, the majority of patients with SMA types 2 and 3 are currently treated with nusinersen, as it was the first therapy to be approved and made available.^40^ Consequently, patients receiving Risdiplam have only recently initiated treatment, which explains the relatively short follow-up period. Furthermore, the study would have been more robust if other motor function scales, biomarkers, and Minimal Clinically Important Difference (MCID) markers had been available to track disease progression.

In conclusion, Risdiplam was shown to be a safe therapeutic option, associated with apparent stabilization or improvement of motor and respiratory functions in most patients with SMA types 2 and 3. Nevertheless, a minority of patients exhibited less favorable outcomes, most commonly in the context of severe osteoskeletal deformities. This early trends need to be confirmed by longer follow-up studies.

## Data availability

The datasets generated and/or analyzed during the current study are available from the corresponding author upon reasonable request.

## Declaration of competing interest

The authors declare no conflicts of interest.
